# Early and frequent exposure to antibiotics in children and the risk of obesity: systematic review and meta-analysis of observational studies

**DOI:** 10.12688/f1000research.24553.1

**Published:** 2020-07-16

**Authors:** Archita Srivastava, Kim Chau, Henry Kwon, Qin Guo, Bradley C. Johnston

**Affiliations:** 1 Department of Medicine, Queen's University, Kingston, Ontario, Canada; 2 Department of Health Research Methods, Evidence & Impact, McMaster University, Hamilton, Ontario, Canada; 3 Department of Medicine, Wayne State University, Detroit, MI, USA; 4 Department of Pediatrics, West China Second University Hospital, Chengdu, China; 5 Department of Nutrition, Texas A&M University, College Station, TX, USA

**Keywords:** Antibiotics, Early Life, Obesity, Overweight, Prenatal

## Abstract

**Background: **This study aimed to systematically evaluate the available evidence on prenatal and early infancy antibiotic exposure and the association with overweight and obesity in later childhood.

**Methods: **We conducted a comprehensive search of Embase, MEDLINE, and Web of Science for observational studies assessing prenatal and early antibiotic exposure on the risk of overweight and obesity. We independently assessed the risk of bias using the ROBINS instrument and the overall quality of evidence using the GRADE approach.

**Results: **Our search identified thirteen observational studies including 554,983 participants; most studies were at moderate risk of bias. We found a statistically significant impact of early antibiotic exposure and the risk of being overweight later in childhood (OR 1.18; 95% CI 1.05 to 1.34) (very low quality evidence). We also found that early childhood antibiotic exposure was associated with the risk for childhood obesity (OR 1.14; 95% CI 1.04 to 1.24) (very low quality evidence).

**Conclusions: **Very low quality evidence suggests that exposure to antibiotics early in life may be associated with an increased risk of being overweight and obese in later childhood.  However, very low quality evidence raises serious questions about the plausibility of prenatal and early infancy antibiotic exposure being causally related to weight in children.

**PROSPERO registration**: CRD42016050011 (14/12/2016)

## Introduction

 Infants and children are commonly and frequently prescribed antibiotics and up to 40% of infants are exposed either directly or through maternal intra-partum antibiotic prophylaxis ^1, 2^. In the United States it is estimated that a child receives approximately three courses of antibiotics by the age of two and 10 courses by the age of 10 years ^3^ and, often, these courses are prescribed for viral infections thus offering no therapeutic benefit ^4^. The concerns with significant overuse of antibiotics are increased antibiotic resistance, increased rates of adverse drug reactions, such as rashes, fungal infections, and antibiotic-associated diarrhea. The intestinal microbiota begins development *in utero* and resembles adult microbiota by 2.5 years of age ^4^. Thus, external exposures to antibiotics during this phase of microbiota development may potentially impact the normal colonization pattern, and the composition of the gut microbiota, both of which may play a role in health outcomes later in life, including weight gain and obesity ^2, 3, 5^.

While the paradigm has been that the infant gut is sterile at birth, increasing evidence suggests that colonization may begin *in utero* with bacterial colonies detected in the placenta and meconium ^6^. Thus, it is possible that prenatal or intrapartum antibiotic exposure may potentially affect these bacterial colonies *in utero*. Most notably, antibiotic exposure can affect the gut microbiota composition at any age; however, infants may especially be vulnerable, as the gut microbiota is highly unstable and dynamic during this period with greater interindividual variability, compared to that of an adult ^7– 9^. Cho *et al.* and Cox *et al.* have shown increased fat accumulation in mice who were treated with antibiotics early in age regardless of antibiotic class ^10– 12^. This corresponds with an absence of certain populations of prominent microbes, such as *Lactobacillus*, *Allobaculum*, *Rikenellaceae,* and *Candidatus Arthromitus due to antibiotic exposure. This suggests a potential protective role of these bacteria against patient-important outcomes, such as weight gain and obesity* ^11, 12^. As such, this paper aims to evaluate and summarize the available evidence on the potential impact of prenatal, intrapartum, and early childhood antibiotic exposure on the risk of overweight and obesity later in life. 

## Methods

This review was registered with PROSPERO - *International Prospective Register of Systematic Reviews* on the 14 ^th^ December 2016 under the number CRD42016050011 ^13^.

### Search strategy

An experienced clinical librarian (TAW) conducted a literature search in the following electronic databases from inception to June 2018: Embase (Ovid), MEDLINE (Ovid), MEDLINE In-Process & Other Non-Indexed Citations (Ovid), MEDLINE ^®^ Epub Ahead of Print (Ovid), and Web of Science. The search terms included database-controlled vocabulary and keywords for the concepts of “ *antibacterial agents*” AND “ *children*” AND “ *microbiome*” OR “ *microbiota*” AND “ *health outcomes*” (e.g. weight, obesity, diabetes). We also supplemented the search by reviewing bibliographies of review articles and other eligible clinical studies to ensure that studies that were not identified by the search strategy were included. Clinicaltrials.gov was searched for unpublished and ongoing trials. No language or date restrictions were applied. See extended data for full search strategies ^14^.

### Study selection

***Inclusion criteria*.** Observational and experimental study designs were eligible, including cohort, cross-sectional, and case-control studies, data modelling studies, and randomized controlled trials. Further, to be eligible, studies had to examine maternal prenatal, intrapartum, and child (birth to 18 years) exposure to antibiotics and at least one of our target health outcomes of interest: overweight (body mass index [BMI] ≥ 25 kg/m ^2^), or obesity (BMI ≥ 30 kg/m ^2^). The comparison group included children that were not exposed to antibiotics. Studies with both fixed and variable follow-up periods were eligible. Studies or subgroups within studies that included premature infants, infants born with low birth weight or comorbidities, such as cystic fibrosis and Crohn’s disease were excluded.

***Screening, data extraction and quality assessment*.** Blinded to the journal of publication and results, two teams of independent reviewers (AS with either LL or HK) screened titles and abstracts of the studies to determine eligibility. Full-text articles were retrieved and assessed for further eligibility assessment. Discrepancies were resolved by discussion and, when necessary, additional input from a third reviewer (BCJ, KC).

Two reviewers (AS with either KC or HK) extracted data independently using a standardized data extraction form. The following data was extracted: study design, study setting, demographic information, antibiotic regimen (frequency and duration of exposures, type of antibiotic/class), and outcome data for each of our target outcomes including reported time points and duration of follow-up.

As no randomized trials were identified, the Risk of Bias In Non-randomized Studies (ROBINS) instrument for different types of observational studies ^15^ was used to assess study validity. Two reviewers (AS and KC) independently evaluated each observational study included for risk of bias (RoB). The ROBINS instrument consists of seven total RoB questions, with two questions under the pre-intervention domain, one question on the intervention domain and four questions under the post-intervention domain ^15^. Response options for each question included ‘low RoB’, ‘moderate RoB’, ‘serious RoB’, and ‘critical RoB’. We modified the instrument; wherein low and moderate risk of bias was classified as ‘low risk of bias’ and serious and critical risk of bias was classified as ‘high risk of bias’. For each study, if one or more questions was judged to be high or critical risk of bias, the overall study was deemed to be at high risk of bias ^15^. Any disagreements regarding data extraction or risk of bias items was resolved through discussion, and, when necessary, with an experienced methodologist (BCJ).

We applied the GRADE (Grading of Recommendations, Assessment, Development, and Evaluations) approach ^16^ for rating the overall quality of evidence for the outcomes of interest. In particular, observational studies were considered low quality evidence, but may be rated up for three reasons: (1) when a large magnitude of effects exists (e.g. OR <0.5 or >2.0), (2) when there is a dose-response gradient or (3) when all plausible confounding or other biases may be working against the observed effect ^16^. Observational studies without these characteristics were considered low quality evidence. In addition, if studies were limited by risk of bias, inconsistency, indirectness, imprecision or publication bias the overall quality of evidence may to rated down to very low quality ^17^. The quality of evidence for each main outcome was determined after considering each of these elements, and categorized as either high (highly confident that the true effect lies close to that of the estimate of the effect); moderate (moderately confident in the effect estimate: the true effect is likely to be close to the estimate of the effect, but there is a possibility that it is substantially different); low (confidence in the effect estimate is limited: the true effect may be substantially different from the estimate of the effect); very low (very little confidence in the effect estimate: the true effect is like to be substantially different from the estimate of effect) ^17^.

### Data analysis

Meta-analysis was conducted using Stata 14 (Stata Corp., College Station, TX, USA). Effect estimates and corresponding confidence intervals were extracted from eligible articles and we calculated the adjusted odds ratio (OR) with corresponding 95% confidence interval with the generic inverse variance method was used to determine relative effects ^18^.

We conducted two primary meta-analyses, one for overweight and one for obesity with obesity including overweight. Random effects models were used due to the anticipated heterogeneity between studies. We used the DerSimonian and Laird method for estimating tau-squared and subsequent adjustment for effect size ^19^.

Heterogeneity was assessed with the I-squared measure and the corresponding Q statistical test ^18^. To further explore heterogeneity, we conducted *a priori* subgroup and sensitivity analyses ^20^. Our three subgroups of interest included (1) the timing of exposure (prenatal versus early exposure), (2) number of antibiotic exposures (1 to 2 versus 3 or more), and (3) time point of outcome assessment (less than 7 years versus 7 years of age or more). Subgroups on timing and number of antibiotic exposures were stated *a priori* ^13^, while the time point of assessment was a *post-hoc* subgroup chosen based on the distribution of outcome assessments among eligible studies. We also conducted a sensitivity analysis on study design limitations (risk of bias). Low and moderate risk of bias was classified as low risk of bias and serious and critical risk of bias was classified as high risk of bias.

## Results

**Figure 1.**
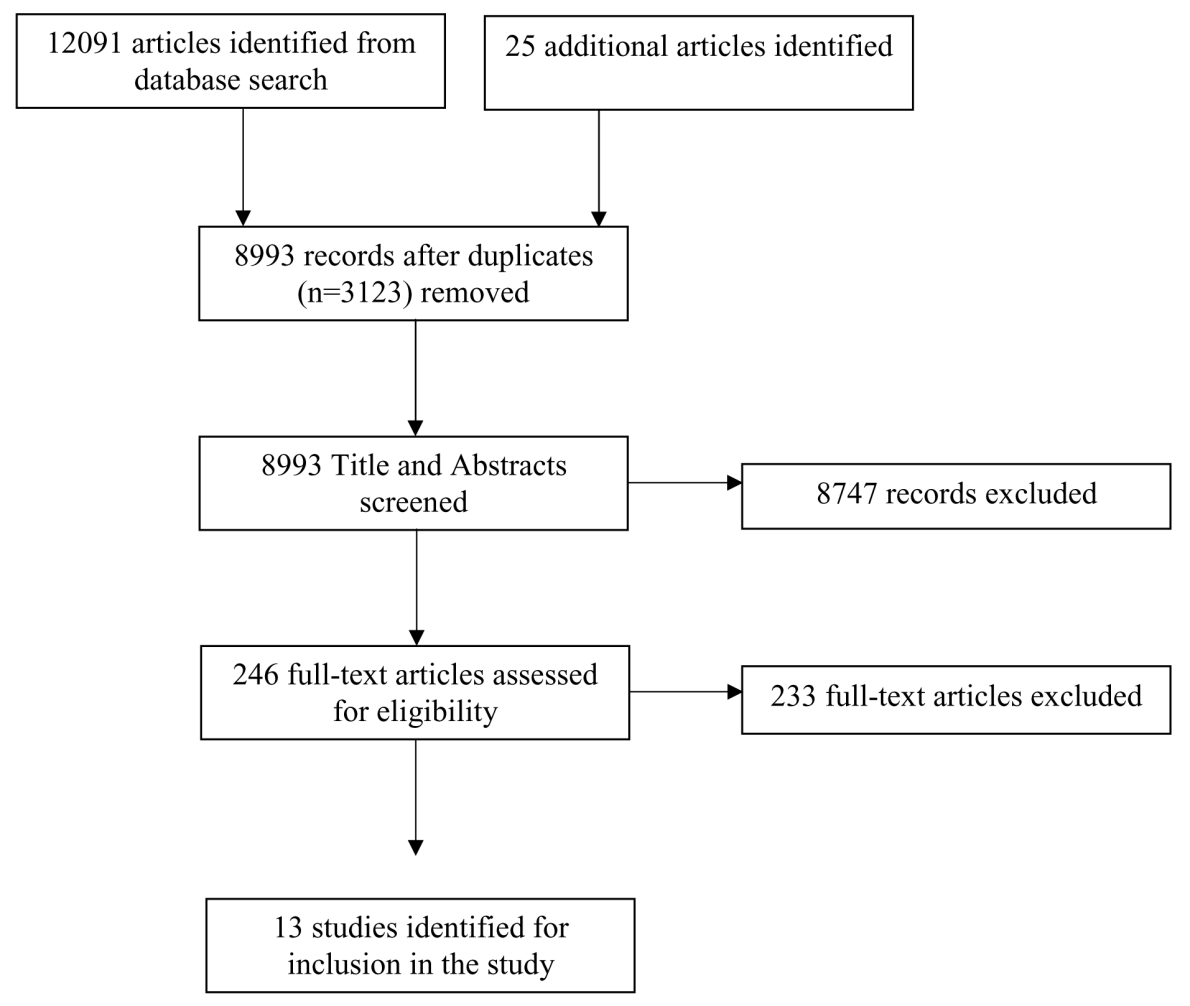
PRISMA flow diagram.

Among 12,091 articles identified, 13 studies were deemed eligible for this review ( Figure 1), including 11 cohort studies ^2, 21– 25, 26– 30^, one cross-sectional study ^31^, and one nested case-control study ^1^. Five studies analyzed the effect of prenatal exposure to antibiotics ^23, 24, 26, 30, 31 ^ while the remaining eight studies evaluated the impact of early childhood (birth to 2 years) antibiotic exposure and the risk of overweight and obesity ^1, 2, 21, 22, 25, 27– 29^. Included studies ranged in size from 97 ^29^ to 312,702 participants ^25^ with a total sample size among all included studies of 554,983. The median age for weight assessment was 7 years (range 2 years to 18 years). A detailed summary of all 13 study characteristics is shown in Table 1.

**Table 1.** Characteristics of studies involving weight-related outcomes.

Study; Country	Study Design	Population	Total Participants Analyzed (% incomplete outcome data)	Population Description	Definition of one antibiotic exposure	Mother, Infant or Both Exposed to Antibiotics	Time- point of Antibiotic Exposure	Weight Outcomes Reported	Time-point of Outcome Assessment	Adjusted Risk Factors
Ajslev *et al.*, 2011; Denmark	Prospective cohort	40,640	28,354 (30.3%)	Danish National Birth Cohort established between 1997–2002	None	Infants	≤6 months old	Overweight: 25≤BMI<30	7 years old	Maternal age, SES, pre- pregnancy BMI, gestational weight gain, smoking, paternal BMI, parity, birth weight, sex, breastfeeding, and age
Azad *et al.*, 2014; Canada	Nested, case- control	723	616 (14.8%)	1995 birth cohort study (Study of Asthma, Genes and the Environment, or SAGE cohort)	Number of prescriptions	Infants	≤1 year old	Overweight: BMI≥85 percentile	9 and 12 years old	Birth weight, breastfeeding, smoke exposure at birth, family income, siblings, diet, physical activity at age 9, current asthma, maternal asthma, and maternal weight
Bailey *et al.*, 2014; USA	Retrospective cohort	89,057	65,480 (26.5%)	Infants in primary care practice network affiliated with the Children’s Hospital of Philadelphia	14-day antibiotic interval	Infants	Ages 0–23 months	Obesity: BMI ≥95 percentile	Ages 24–59 months	Demographic, medical practice, and clinical covariates (parental obesity, SES, early diet and lifestyle)
Bridgman *et al.*, 2018, Canada	Prospective cohort	988	891 (9.9%)	Sub-cohort of the Canadian Healthy Infant Longitudinal Development (CHILD) study	None	Mothers	2 ^nd^ and 3 ^rd^ trimester of pregnancy	Overweight: Weight for length z score>97 percentile	1 year	mode of delivery, breastfeeding status and infant antibiotic exposure
Cassidy- Bushrow *et al.*, 2018; USA	Prospective cohort	659	527 (20%)	Pregnant women who delivered from September 2003 through December 2007, and part of Wayne County Health, Environment, Allergy, and Asthma cohort (WHEALS)	None	Mothers	1 ^st^, 2 ^nd^ and 3 ^rd^ trimester of pregnancy	Overweight: BMI ≥85 percentile	2 years old	Infant gender, delivery mode, ever breastfed, maternal age, maternal race, maternal education, birthweight Z- score, child age at 2-year visit and BMI at first prenatal care visit
Li *et al.*, 2017, USA	Prospective cohort	312, 702	260,556 (16.7%)	Infants delivered between 1997 to 2013 who were a part of Kaiser Permanente Northern California (KPNC) member population	Number of prescriptions	Infants	≤24 months old	Obesity: BMI ≥95 percentile	18 years old	Infection type and severity (ie, number of infection episodes), maternal age, race or ethnic origin, pre- pregnancy BMI, preterm delivery, low birthweight, maternal antibiotic use, and infection during pregnancy
Mor *et al.*, 2015; Denmark	Cross- sectional	9,886	9,886 (0%)	Infants born between 1994 and 1998 in Aalborg municipality who underwent routine school anthropometric evaluation	Number of prescriptions	Mothers	1 ^st^, 2 ^nd^ and 3 ^rd^ trimester of pregnancy	Overweight: 25≤BMI<30 Obesity: BMI ≥30	7–16 years old	Maternal age at delivery, marital status, smoking during pregnancy and multiple gestations
Mueller *et al.*, 2015; USA	Prospective cohort	727	436 (40%)	Mothers enrolled in the Northern Manhattan Mothers and Children Study	None	Mothers	2 ^nd^ and 3 ^rd^ trimester of pregnancy	Obesity: BMI ≥95 percentile	7 years old	Offspring sex, ethnicity, birth weight, maternal age, maternal pre-gravid BMI, receipt of public assistance during pregnancy, delivery mode and breastfeeding
Saari *et al.*, 2015; Finland	Retrospective cohort	14,764	12,062 (18.3%)	Infants of the Finnish growth reference study population born between 2003 and 2007 who attended child welfare clinics in the city of Espoo, Finland	Number of episodes	Infants	≤24 months old	Overweight: 25≤BMI<30	2 years old	Maternal smoking after first trimester, parental relationship, mode of delivery, birth weight, and birth length
Scott *et al.*, 2016; United Kingdom	Retrospective cohort	26,867	21,714 (19.2%)	Infants born between 1995 to 2013 who are part of The Health Improvement Network (THIN).	None	Infants	<24 months old	Obesity: BMI z-score ≥2.37 for males and ≥2.25 for females	4 years old	Year of birth, maternal and sibling obesity, maternal diabetes, mode of delivery, country of origin, urban environment, and Townsend score
Trasande *et al.*, 2013; United Kingdom	Prospective cohort	14,541	11,532 (20.7%)	Infants part of Avon Longitudinal Study of Parents and Children (ALSPAC) born between 1991–1992	None	Infants	<6 months, 6–14 months, 15–23 months	Obesity: BMI ≥95 percentile, BMI z score (38mo, 7y) Overweight: BMI: 85 to 94 percentile (38mo, 7y)	Overweight, Obesity: 38 months and years	Birth weight, maternal parity, race, social class, education, parental BMI, first trimester smoking, breastfeeding, timing of introduction of complementary foods, time spent per day watching television, in car on weekdays /weekends, and dietary pattern classifications
Ville *et al.*, 2017; USA	Prospective cohort	97	74 (23.7%)	Pregnant, self-identified Latina women seen in prenatal clinics at San Francisco General Hospital (SFGH) in 2012 and 2013	None	Infants	6 months	Obesity: BMI ≥95 percentile	2 years	Maternal BMI, weight for length z-score at birth, breastfeeding at 6 months, rapid infant weight gain, infant sex
Wang *et al.*, 2018; USA	Prospective cohort	43,332	39,615	Mothers part of Perinatal Collaborative Project who gave birth between 1959–1965	Antibiotic courses	Mothers	1 ^st^, 2 ^nd^ and 3 ^rd^ trimester of pregnancy	Overweight: BMI ≥95 percentile Obesity: BMI ≥95 percentile	4 years old, 7 years old	Maternal age, race, SES, smoking, pre- pregnancy BMI

SES = socioeconomic status; BMI = body mass index

### Risk of bias assessment

Using ROBINS instrument to independently assess risk of bias for weight-related outcomes, 11 studies were rated as ‘moderate’ for risk of bias ^1, 21– 25, 27– 30, 31^ and two studies were rated as ‘serious’ for risk of bias ^2, 26^. The studies rated as serious risk of bias were missing significant participant outcome data, with exclusion of participants introducing bias in the study. Despite all studies having adjusted for potential confounders, each included study was rated as moderate risk of bias for confounding. The choice and number of variables adjusted for differed considerably between studies. In some instances, the adjustment factors were theoretically not potential confounders (e.g. ethnicity), while in other instances, potential confounders such as socioeconomic status were not adjusted for. The complete risk of bias assessment for each individual study is provided in Table 2.

**Table 2.** ROBINS-I Risk of Bias Assessment.

Study	Pre-intervention (baseline)		At intervention	Post-intervention				Overall RoB Judgement
	Bias due to Confounding ^a^	Bias in Selection of Participants ^b^	Bias in Measurement of Interventions ^c^	Bias Due to Departures from Intended Interventions ^d^	Bias Due to Missing Data ^e^	Bias in Measurement of Outcomes ^f^	Bias in Selection of Reported Results ^g^	
Ajslev, 2011	Moderate	Moderate	Moderate	Low	Moderate	Moderate	Moderate	Moderate
Azad, 2014	Moderate	Moderate	Moderate	Moderate	Moderate	Low	Moderate	Moderate
Bailey, 2014	Moderate	Low	Moderate	Low	Moderate	Moderate	Low	Moderate
Bridgman, 2018	Moderate	Low	Low	Moderate	Low	Low	Low	Moderate
Cassidy- Bushrow, 2018	Moderate	Moderate	Low	Moderate	Low	Low	Low	Moderate
Li, 2017	Moderate	Moderate	Low	Moderate	Low	Low	Moderate	Moderate
Mor, 2015	Moderate	Moderate	Low	Moderate	Moderate	Low	Moderate	Moderate
Mueller, 2015	Moderate	Moderate	Moderate	Moderate	Serious	Moderate	Moderate	Serious
Saari, 2015	Moderate	Moderate	Low	Moderate	Moderate	Moderate	Low	Moderate
Scott, 2016	Moderate	Moderate	Moderate	Moderate	Moderate	Moderate	Low	Moderate
Trasande, 2013	Moderate	Moderate	Moderate	Moderate	Serious	Moderate	Moderate	Serious
Ville, 2017	Moderate	Moderate	Moderate	Moderate	Moderate	Low	Moderate	Moderate
Wang, 2018	Moderate	Moderate	Low	Moderate	Moderate	Moderate	Moderate	Moderate

^a^ Confounding: One or more prognostic variables also predicts the intervention received at baseline. ^b^ Selection Bias: When exclusion of some eligible participants is related to both intervention and outcome, there will be an association between interventions and outcome even if the effect of interest is truly null. ^c^ Information Bias: Bias introduced by either differential or non-differential misclassification of intervention status. ^d^ Confounding: Bias that arises when there are systematic differences between experimental intervention and comparator groups in the care provided, which represent a deviation from the intended intervention. ^e^ Selection Bias: Bias that arises when later follow-up is missing for individuals initially included and followed (e.g. differential loss to follow-up that is affected by prognostic factors) ^f^ Information Bias: Bias introduced by either differential or non-differential errors in measurement of outcome data. Such bias can arise when outcome assessors are aware of intervention status, if different methods are used to assess outcomes in different intervention groups, or if measurement errors are related to intervention status or effects. ^g^ Reporting Bias: Selective reporting of results from among multiple measurements of the outcome, analyses or subgroups in a way that depends on the findings.

### Overweight

**Figure 2.**
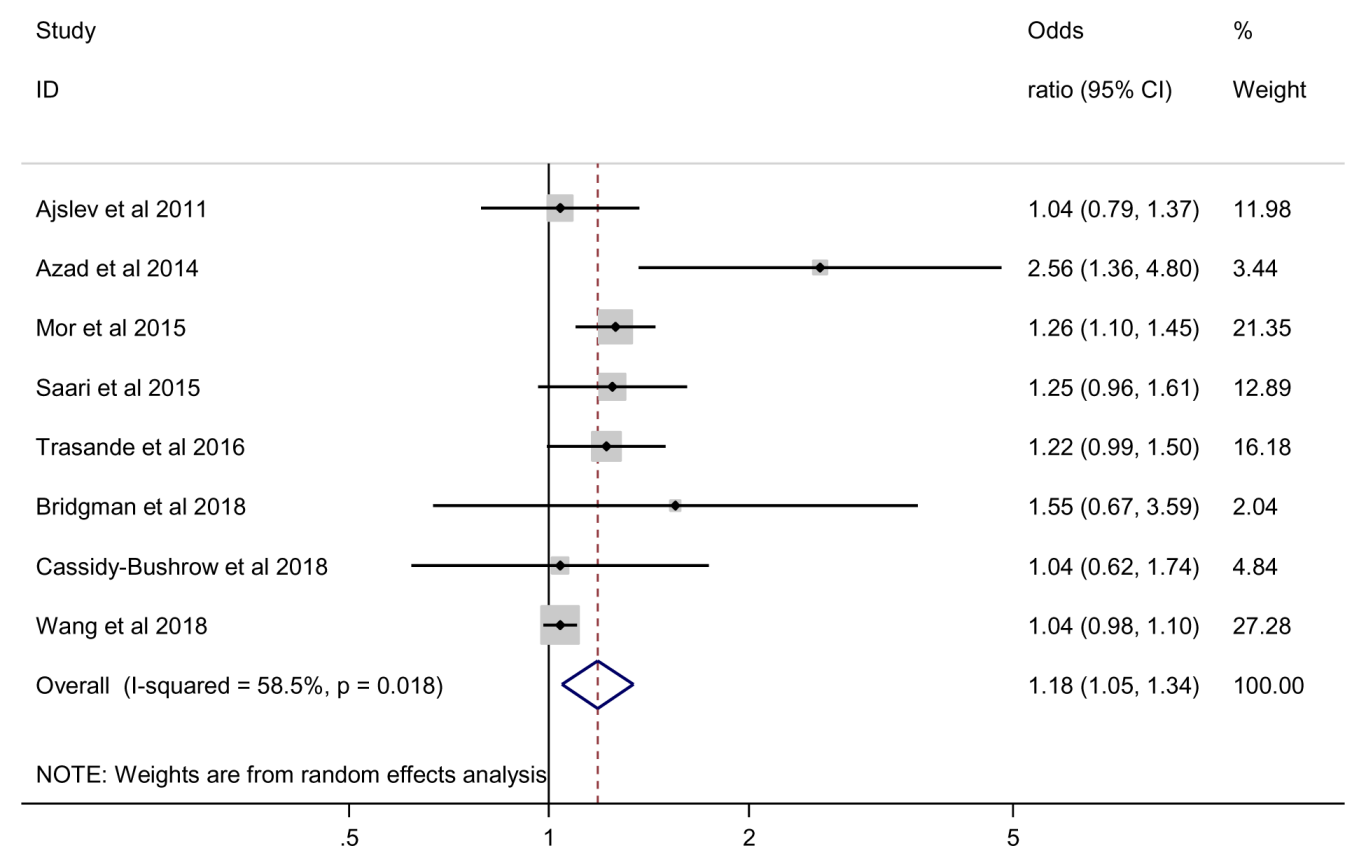
Overweight outcome.

We found statistically significant effect of antibiotics on the risk of being overweight (OR 1.18; 95% CI 1.05 to 1.34, 8 studies, 125,533 participants, I ^2^=58.5%) ( Figure 2). Using the estimated population risk of being overweight ^32^, the risk difference between exposed and non-exposed was equivalent to 26 more overweight cases per 1000 children (95%CI 7 more to 47 more cases). Our GRADE assessment indicates that the overall quality of evidence for the risk of being overweight was very low due to serious indirectness ( Table 3).

**Table 3.** GRADE summary of findings.

Early and frequent exposure to antibiotics in children and the risk of weight gain and obesity							
**Patient or population:** Children with early and frequent exposure to antibiotics **Settings:** Children followed in observational studies, primarily prospective cohorts **Exposure:** Antibiotics **Non-exposure:**No antibiotics							
**Outcomes**	**Illustrative comparative risks** * (95% CI)			**Relative** **effect** **(95%** **CI)**	**No of Participants** **(studies)**	**Quality of the** **evidence (GRADE)**	**Comments**
	**Baseline** **risk**	**Corresponding risk**					
	**Risk with** **Control**	**Risk with** **early** **exposure** **to** **antibiotics**	**Risk** **Difference** **(95% CI)**				
**Overweight**	**180 per** **1000** ^1^	**206 per** **1000** (187 to 227)	26 more overweight or obese cases per 1000 (7 more to 47 more)	**OR 1.18** (1.05 to 1.34)	n = 125, 533 (8 studies)	**Very Low** ^2– 8^	
**Obesity**	**70 per** **1000** ^9^	**79 per** **1000** (73 to 85)	9 more obese cases per 1000 (3 more to 15 more)	**OR 1.14** (1.04 to 1.24)	n = 497, 209 (8 studies)	**Very Low** ^5– 8. 10– 12^	
*The basis for the **assumed risk** (e.g. the median control group risk across studies) is provided in footnotes. The **corresponding risk** (and its 95% CI) is based on the assumed risk in the comparison group and the **relative effect** of the intervention (and its 95% CI). **CI:** Confidence interval; **RD:** Risk difference; **OR:** Odds ratio							
GRADE Working Group grades of evidence **High quality:** Further research is very unlikely to change our confidence in the estimate of effect. **Moderate quality:** Further research is likely to have an important impact on our confidence in the estimate of effect and may change the estimate. **Low quality:** Further research is very likely to have an important impact on our confidence in the estimate of effect and is likely to change the estimate. **Very low quality:** We are very uncertain about the estimate.							

^1^ This control group estimate is the risk of being overweight or obese in children 5 to 19 years old. The risk estimate comes from the WHO website ( https://www.who.int/news-room/fact-sheets/detail/obesity-and-overweight). ^2^ Seven studies were judged to be moderate risk of bias, while one was judged to be serious risk of bias and we did not downgrade for study limitations ^3^ Heterogeneity among eight studies (P = 0.018, I ^2^ = 58.5%) was considered to be moderate according to the Cochrane Handbook (2011). All studies however had over lapping 95%CIs and demonstrated the same direction of effect. We therefor chose to not downgrade. ^4^ With respect to indirectness issues, participants exposed were different, wherein 4 studies included infants (post-natal), while the other 4 studies included mothers (pre-natal) as the population exposed to antibiotics. In our subgroup assessment of post-natal versus pre-natal, we found no significant difference between groups indicating limited evidence to suggest that timing of exposure impacts weight (P = 0.518). With respect to timing of assessment, there were different time points among 8 studies, ranging from 1 year age to 16 years of age. Our subgroup analysis on the timing of assessment, although dichotomized due to lack of power (<7 years versus ≥ 7 years), showed no significant difference (P = 0.699). With respect to measuring weight, there were 5 different definitions (e.g. BMI from 25 to <30; BMI > 85 percentile) of documenting weight among 8 studies. We decided to downgrade for serious indirectness related to the measurement of weight, particularly because we could not conduct an appropriate subgroup analysis (i.e. conduct meta-regression or dichotomize). ^5^ The results are precise and we did not downgrade for imprecision. ^6^ Given there were fewer than 10 studies we could not assess for publication bias. ^7^ With respect to the size of the effect related to antibiotic exposure, all studies consistently demonstrating an odds ratio of < 2 and we did not upgrade. ^8^ In our subgroup assessment of those receiving 1 to 2 antibiotic exposures versus those receiving 3 or more, we found no evidence of an increased risk with an increased dose and we therefor did not rate up for dose response. ^9^The risk estimates come from WHO website ( https://www.who.int/news-room/fact-sheets/detail/obesity-and-overweight) and the Non-Communicable Diseases (NCD) Risk Factor Collaboration (Lancet 2017 Dec 16;390(10113):2627-2642). ^10^ Six of eight studies were judged to be at moderate risk of bias overall risk of bias and we did not downgrade for study limitations. ^11^ Substantial heterogeneity among studies (P < 0.001, I ^2^ = 76.8%). Although all studies have same direction of effect, the 95%CIs do not fully overlap. Further, our subgroup analysis did not explain the observed heterogeneity and hence we downgraded. ^12^ With respect to indirectness issue, participants exposed were different, wherein 5 studies included infants (post-natal), while the other 3 studies included mothers (pre-natal) as the population exposed to antibiotics. In our subgroup assessment of post-natal versus pre-natal, we found no significant difference between groups indicating limited evidence to suggest that timing of exposure impacts weight (P = 0.353). With respect to timing of assessment, there were different time points among 8 studies, ranging from 2 years age to 16 years of age. Our subgroup analysis on the timing of assessment, although dichotomized due to lack of power (< 7 years versus ≥ 7 years), showed no significant difference (P = 0.853). With respect to measuring the potential impact of antibiotics on weight, there were 3 different definitions (e.g. BMI ≥30; BMI > 95 percentile) of documenting weight among 8 studies and the reference control group was both normal weight, normal weight plus overweight, and we rated down for indirectness issues.

### Obesity

**Figure 3.**
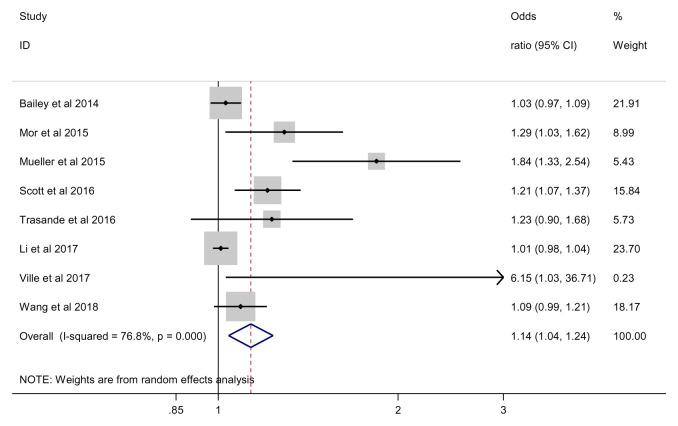
Obesity outcome.

We found an overall significant effect of antibiotics on the risk of obesity (OR 1.14; 95% CI 1.04 to 1.24, 7 studies, 497,209 participants, I ^2^=76.8%) ( Figure 3). Based on the estimated population risk of obesity in children ^14^, the risk difference between those exposed and non-exposed to antibiotics was equivalent to 9 more obesity cases per 1000 (95%CI 3 more to 15 more cases). Again, our GRADE assessment indicated that the overall quality of evidence for the obesity was very low due to serious inconsistency between studies ( Table 3).

### Subgroup and sensitivity analysis for overweight outcome

**Figure 4.**
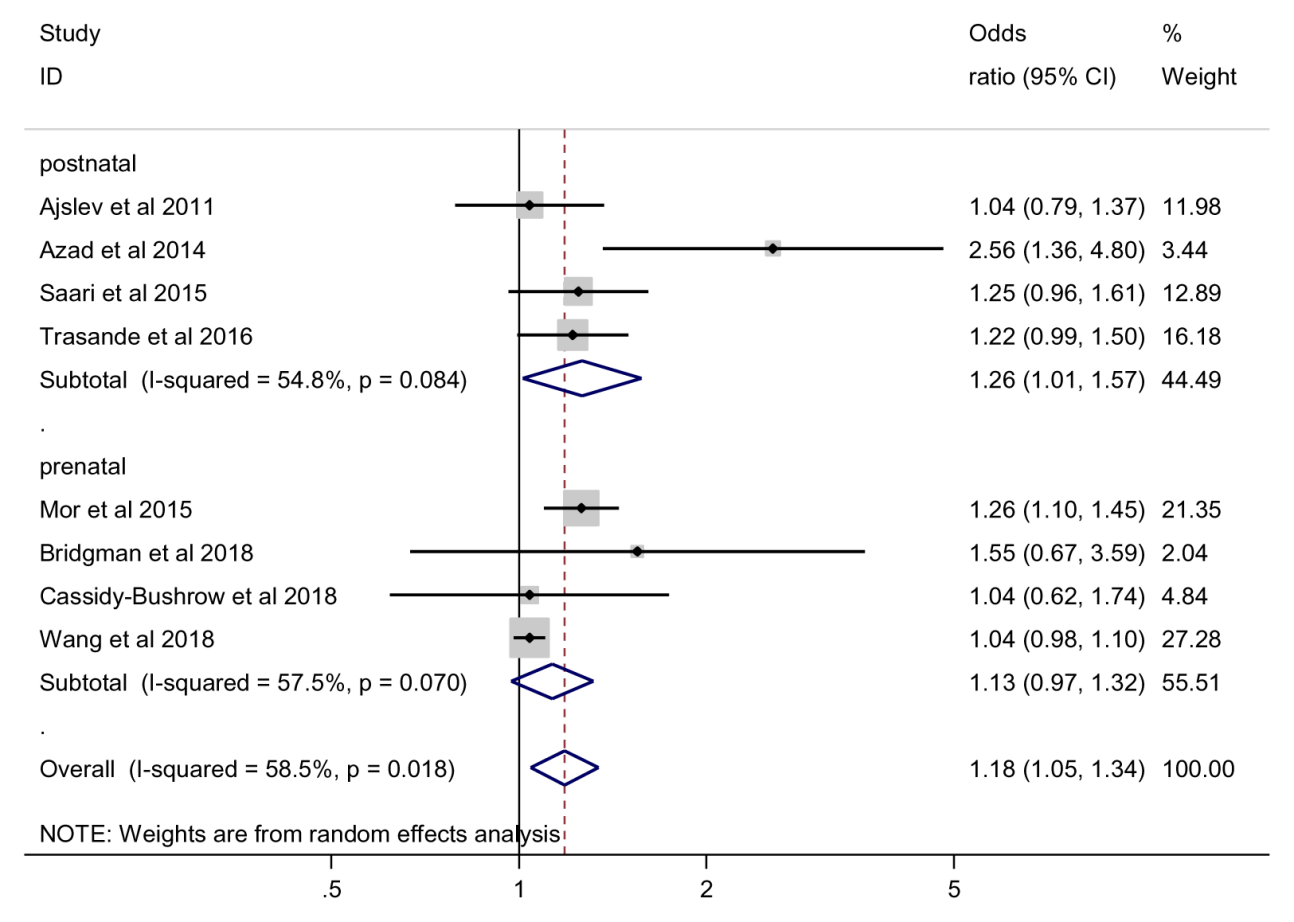
Overweight outcome, subgroup analysis by timing of exposure.

***Prenatal vs. early infancy antibiotic exposure*.** For prenatal timing of exposure, the effect of antibiotics on the risk of being overweight was not statistically significant, random effects (OR = 1.13, 95% CI 0.97 to 1.32, 4 studies, 54865 participants, I ^2^=57.5%) ( Figure 4). For early infancy exposure, we found a significant effect of antibiotics on the risk of being overweight (OR = 1.26, 95% CI 1.0 to 1.57, 4 studies, 70668 participants, I ^2^=54.8%). Our meta-regression analysis showed no significant difference between subgroups with regard to the effect of antibiotics on the risk of being overweight outcome (p =0.518).

**Figure 5.**
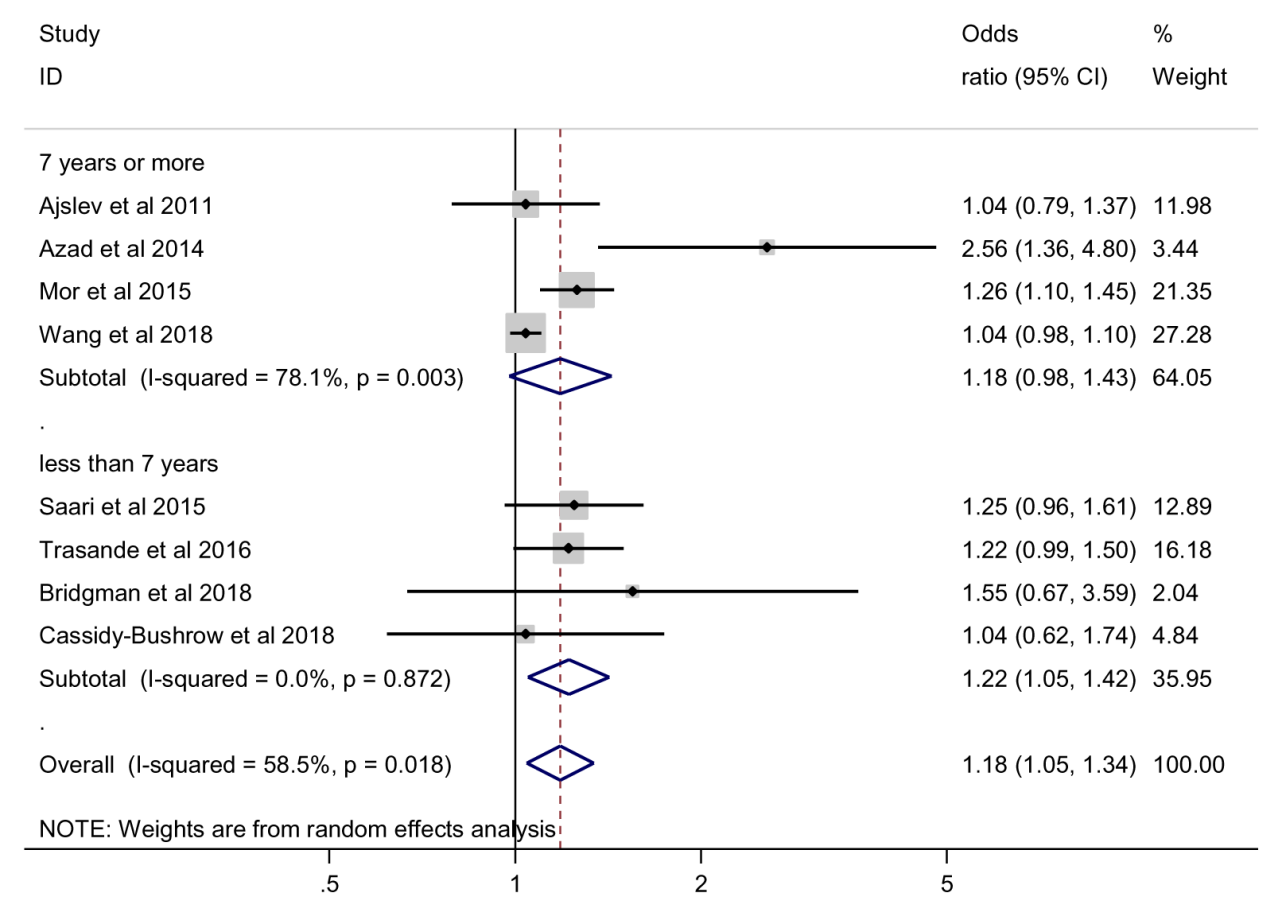
Overweight outcome, subgroup analysis by time-point of outcome assessment.

***Outcome assessment based on follow-up time point*.** When the risk was assessed among those less than 7 years, we found a statistically significant effect of antibiotics on this risk of being overweight (OR = 1.22, 95% CI 1.05 to 1.42, 4 studies, 30952 participants, I ^2^=0%). When the risk was assessed at 7 years or more, the effect of antibiotics on the risk of being overweight was not statistically significant, random effects OR = 1.18, 95% CI 0.98 to 1.43, 4 studies, 94581participants, I ^2^=78.1%) ( Figure 5). Meta-regression analysis showed no significant difference between subgroups with regards to the effect of antibiotics on the risk of being overweight (p = 0.699).

**Figure 6.**
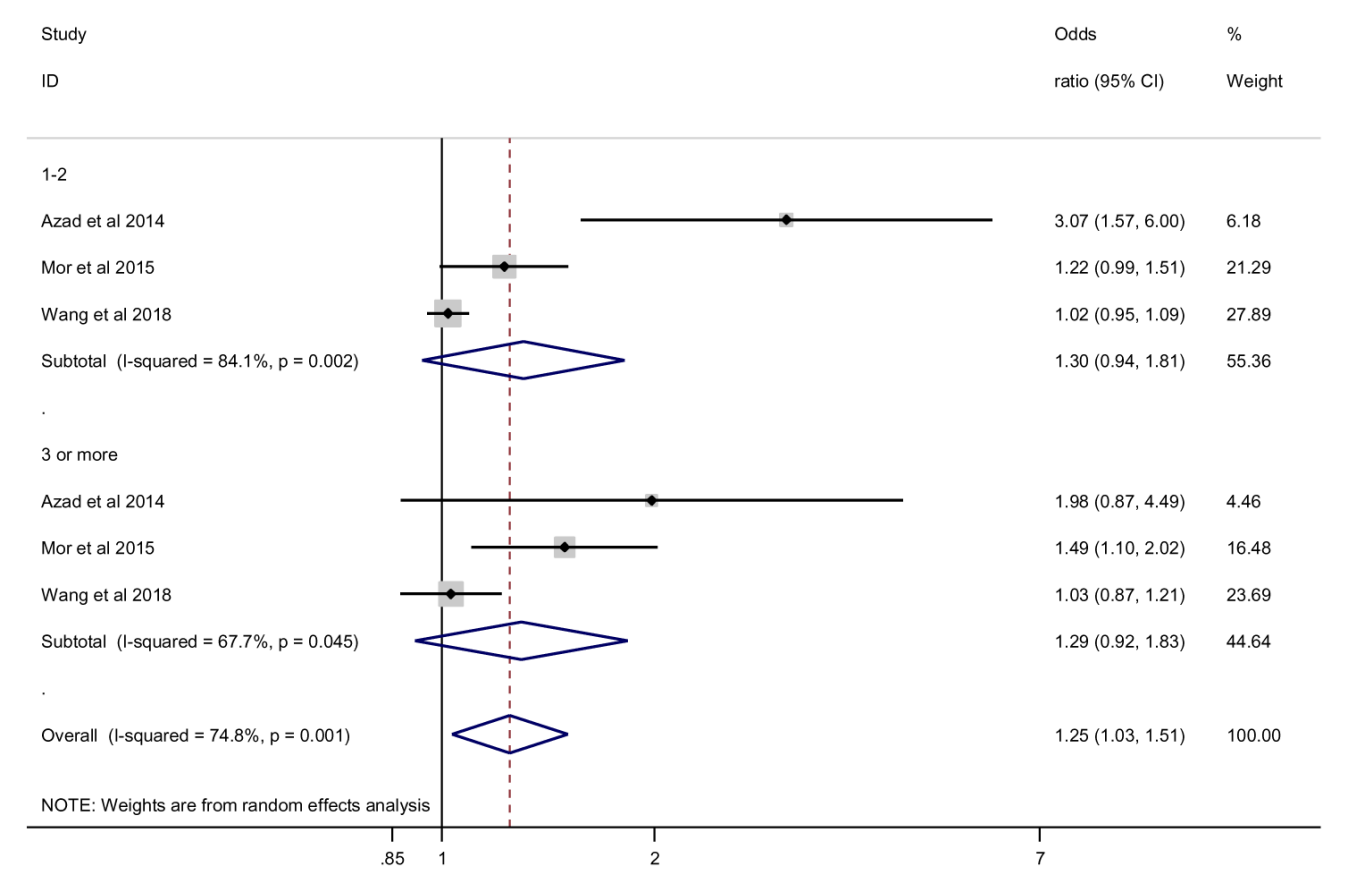
Overweight outcome, subgroup analysis by number of antibiotic exposures.

***Number of antibiotic exposures*.** For 1 to 2 antibiotic exposures, the effect of antibiotics of antimicrobials on the risk of being overweight was not statistically significant (OR = 1.30, 95% CI 0.94 to 1.81, 3 studies, 53941 participants, I ^2^=84.1%) ( Figure 6). For 3 or more antibiotic exposures, the effect of antimicrobials on the risk of being overweight was not statistically significant, random effects (OR = 1.29, 95% CI 0.92 to 1.83, 3 studies, 53941participants, I ^2^=67.7%). Similarly, our meta-regression analysis showed no significant difference between subgroups for antibiotic exposure and overweight risk (p =0.937).

**Figure 7.**
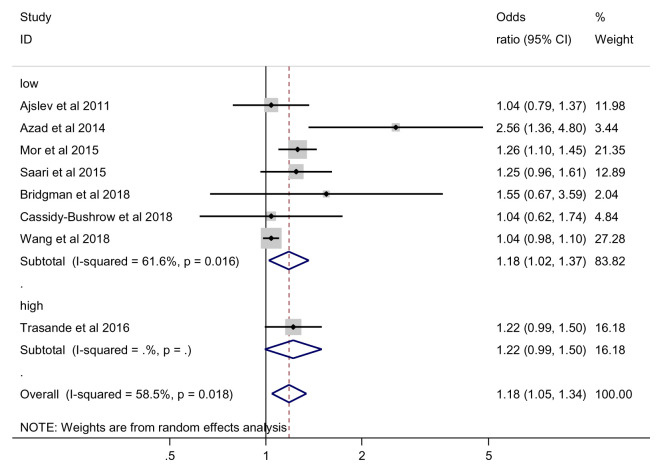
Overweight outcome, sensitivity analysis by risk of bias.

***Sensitivity analysis: Risk of bias assessment*.** For low risk of bias studies, we found a statistically significant effect of antibiotics on the risk of being overweight (OR = 1.18, 95% CI 1.02 to 1.37, 7 studies, 110992 participants, I ^2^=61.6%) ( Figure 7).

### Subgroup and sensitivity analysis for obese outcome

**Figure 8.**
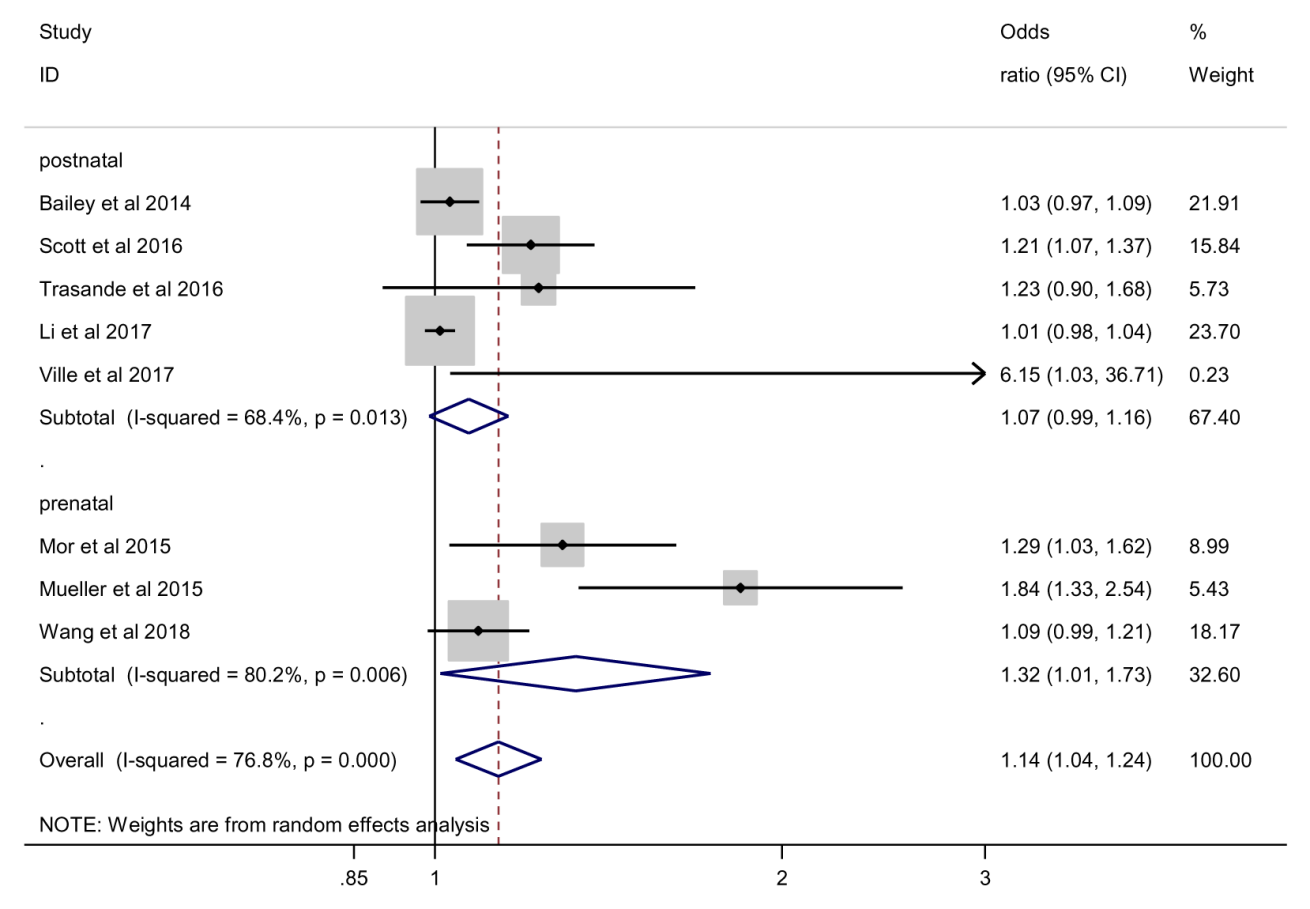
Obesity outcome, subgroup analysis by timing of exposure.

***Prenatal vs. early antibiotic exposure*.** For prenatal timing of exposure, we found a significant effect of antibiotics on obesity (OR 1.32; 95% CI 1.01 to 1.73, 3 studies, 53,945 participants, I ^2^=80.2%) and a non-significant effect for early infancy exposure (OR 1.07; 95% CI 0.99 to 1.16, 5 studies, 443,264 participants, I ^2^=68.4%) ( Figure 8). However, subgroup analysis showed no significant difference between prenatal and early infancy subgroups on the risk of obesity (p = 0.353).

**Figure 9.**
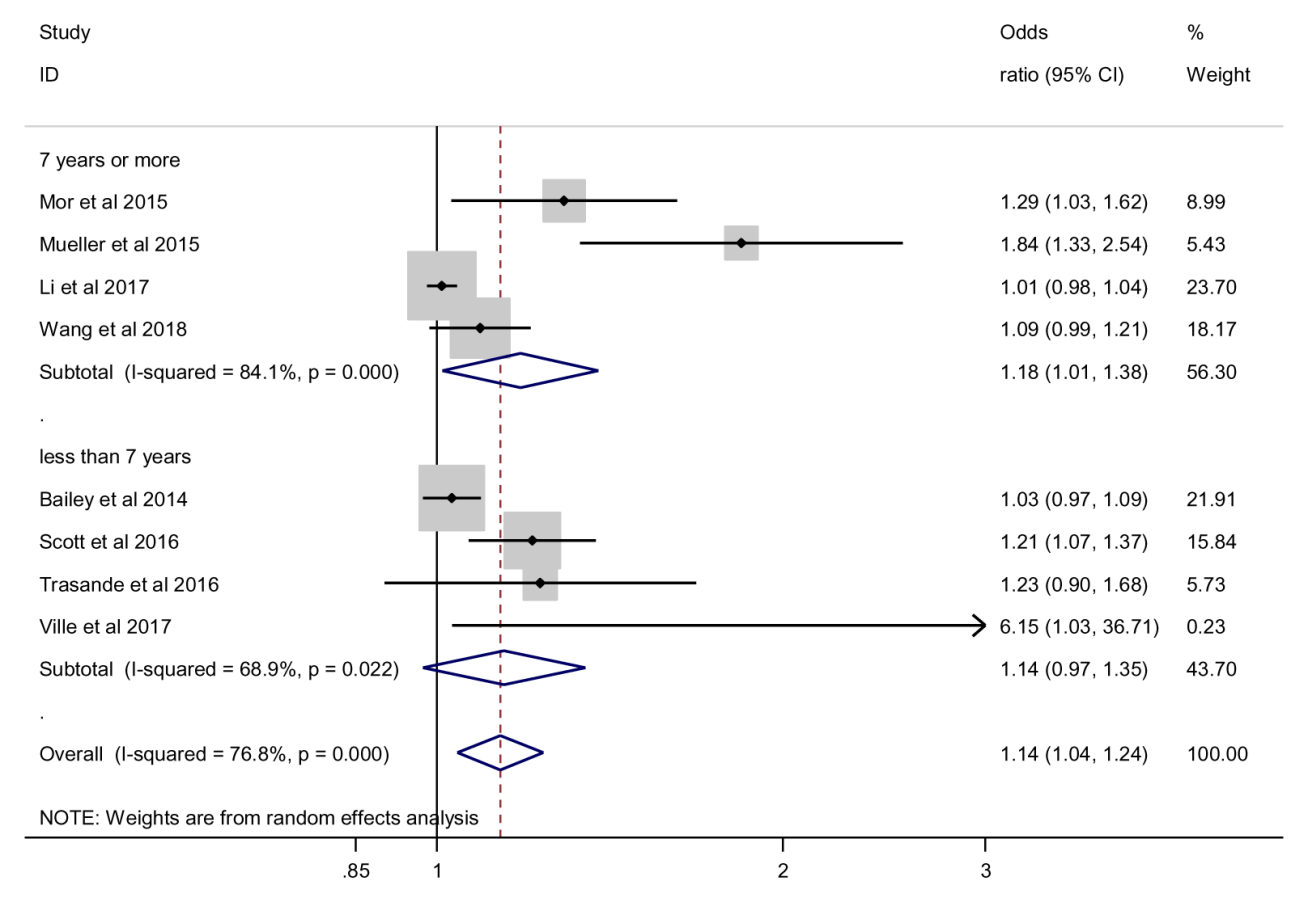
Obesity outcome, subgroup analysis by time-point of outcome assessment.

***Timepoint of outcome assessment*.** For the time point of outcome assessment (less than 7 years), the effect of antibiotics on obesity risk was not statistically significant (OR 1.14, 95% CI 0.97 to 1.35, 4 studies, 130,562 participants, I ^2^=68.9%), while for 7 years or more, we found a significant effect (OR 1.18, 95% CI 1.01 to 1.38, 4 studies, 366,647 participants, I ^2=^84.1%) ( Figure 9). Subgroup analysis showed no significant difference between subgroups (p = 0.853).

**Figure 10.**
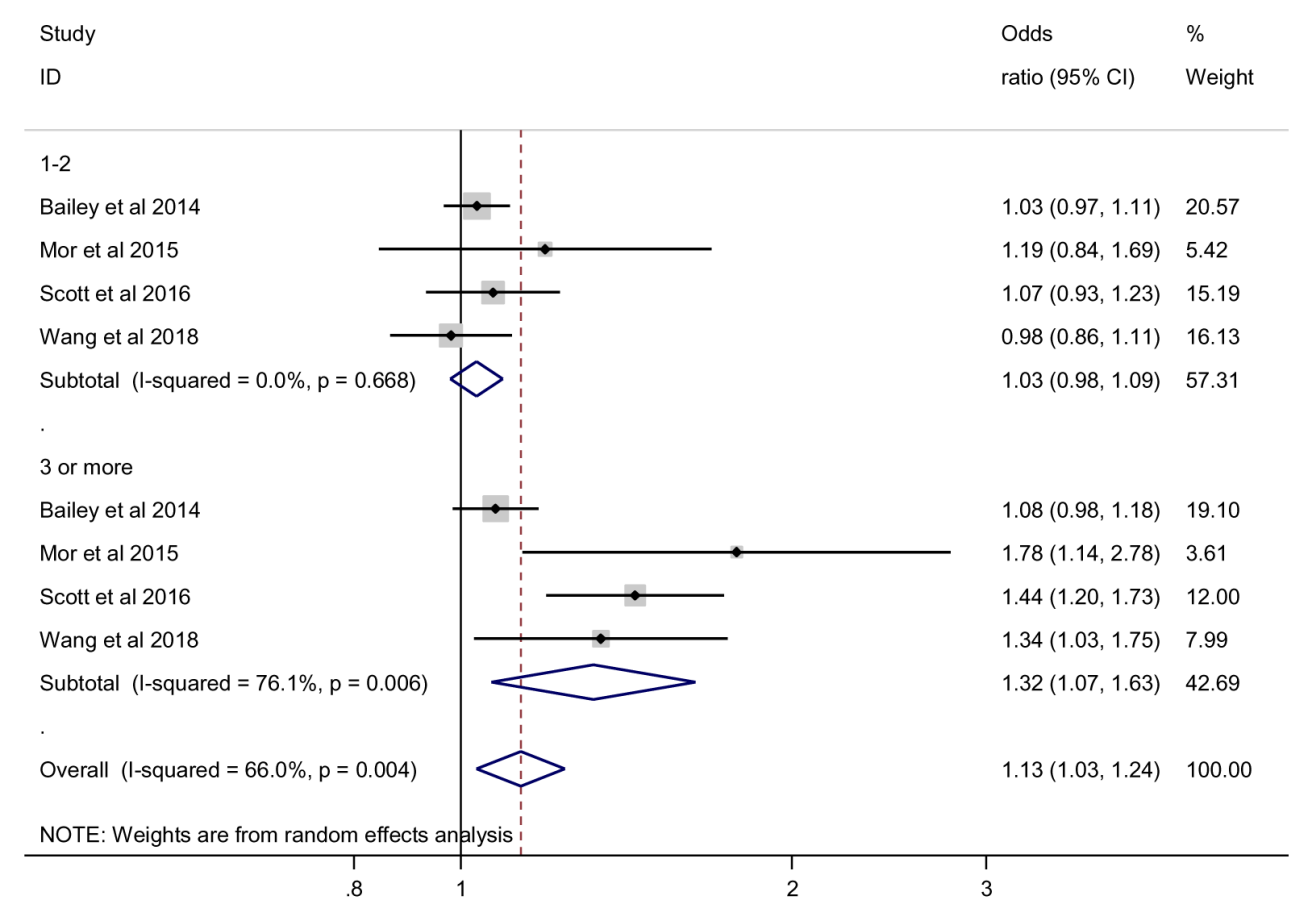
Obesity outcome, subgroup analysis by number of antibiotic exposures.

***Number of antibiotic exposures*.** For those with 1 to 2 antibiotic exposures, the risk of obesity was not statistically significant (OR 1.03, 95% CI 0.98 to 1.09, 4 studies, 70,199 participants, I ^2^=0%), while for those with 3 or more exposures, we found no statistically significant effect (OR 1.29, 95% CI 0.92 to 1.83, 4 studies, 70,199 participants, I ^2^= 76.1%) ( Figure 10). Again, subgroup analysis showed no significant difference between 1 to 2 exposures and 3 or more exposures (p = 0.085). The subgroup results, however, reached statistical significance with 3 or more antibiotics having a larger associated OR for obesity later in life.

**Figure 11.**
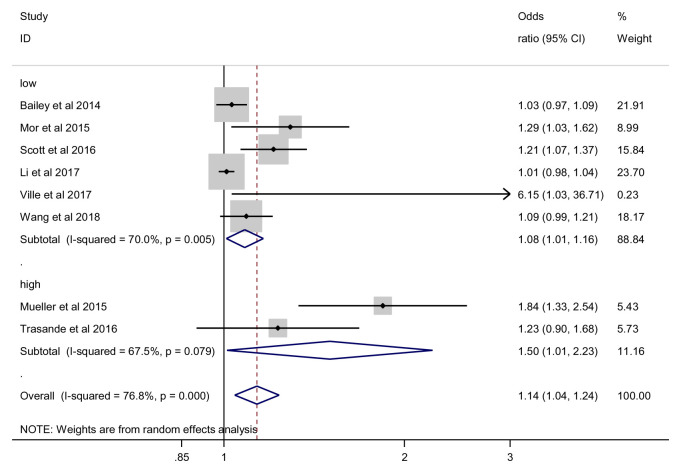
Obesity outcome, sensitivity analysis by risk of bias.

***Sensitivity analysis: Risk of Bias assessment*.** For low (6 studies, 48,1941 participants) versus high risk of bias (2 studies, 15268 participants) studies, we found no significant difference between subgroups (p = 0.118) ( Figure 11).

## Discussion

In total, 13 observational studies with over 554,983 participants were identified that examined the association between prenatal or early childhood antibiotic exposure and the risk of weight-related outcomes. Overall, based on very low quality evidence, antibiotic exposure may be associated with overweight and obesity. The absolute risk increase between those exposed and non-exposed to antibiotics was equivalent to 26 more overweight cases per 1,000 children followed, and 9 more obesity cases per 1,000 children followed.

We specified *a priori* subgroup hypotheses to assess the stability of association across population and intervention variables thought to potentially modify our outcomes of interest. We found a statistically significant association between prenatal antibiotic exposure and obesity, between late antibiotic exposure (>7 years) and obesity and between higher frequency of exposures to antibiotics (3 or more scripts) and the risk of obesity. When we conducted a test of interaction for each of our subgroups, although none were statistically significant, the frequency of exposure was almost significant (p = 0.085), suggesting that higher antibiotic frequency may increase the risk of obesity later in life. This finding corresponds to our *a priori* hypothesis ^13^ that with higher exposure comes a higher risk of obesity, potentially attributable to a more frequent disruption of the composition of the gut microbiota ^7– 9^.

The quality of evidence for each outcome was determined using the GRADE criteria ^17^. For the primary outcome, overweight, the quality of evidence was categorized as very low because of serious indirectness related to the measurement of weight, there were 5 different definitions (e.g. BMI from 25 to <30; BMI > 85 percentile). For the obesity health outcome, the quality of evidence was categorized as very low due to indirectness (the reference control group was both normal weight and normal weight plus overweight) and inconsistency between studies (P < 0.001, I ^2^ = 76.8%), and our subgroup analyses did not explain the observed heterogeneity. Despite authors’ adjustment for a variety of known or suspected confounders, observational studies are prone to residual confounding and after considering each of the GRADE criteria, including the possibility of rating up for magnitude of effect and for dose-response, the evidence provides only very low quality evidence.

The association between antibiotic exposure in early life and increased risk of being overweight or obese is also supported by observations from animal studies. A study conducted by Angelakis *et al.* (2013) ^33^ reported an increase in fat accumulation in mice when the mice were exposed to antibiotics at the weaning stage. The underlying mechanisms may be due to an upregulation of genes involved in lipogenesis, as well as antibiotic-induced changes in gut microbiota composition resulting in increased production of short-chain fatty acid, an additional energy source which results in imbalance of energy regulation, contributing to obesity ^34, 35^. In other studies, it has been suggested that microbial colonization may begin *in utero* through exchange of placental bacteria from mother to fetus; thus, placental transfer of antibiotics consumed during pregnancy may enter fetal circulation resulting in microbial changes similar to that of early life antibiotic exposure ^10, 36^.

Our paper has several strengths. First, our review included a systematic literature search of three primary databases and gray literature sources and is the most comprehensive review to date including 13 studies on weight-related outcomes. Second, an *a priori* design was published as a written protocol and registered with PROSPERO ^13^. We closely followed our *a priori* protocol, however, we did make some *post-hoc* analysis and presentation decisions including the addition of a subgroup on follow-up time (up to 6 years vs 7 or more years of age). Third, we independently assessed the quality of the evidence for each outcome using the GRADE approach, allowing us to document the uncertainty we have in attributing antibiotic exposure as a causal risk factor. Fourth, we quantitatively analyzed our *a priori* subgroups of interest including prenatal versus early infancy antibiotic exposure, and frequency of exposure to explore hypothesized effect modifiers ^1, 22, 26^, suggesting based on the available data, that frequency of exposure deserves further study.

 This systematic review also has two major limitations. First, the search for included articles in this review was completed in June 2018. However, the discussion (below) has been updated to include recent evidence from studies published since spring 2018. Second, there was significant heterogeneity across the included studies with respect to the number and types of antibiotics used, the timeframe between antibiotic exposure and weight assessment, and the method of weight assessment. Although we minimized confounding by using the most adjusted analyses from each study in our meta-analyses, spurious results due to residual confounding remains a plausible explanation for all associations. That is, given that all the included studies were observational in nature, there is a risk of uncontrolled confounding factors despite multivariable adjustment ^37^. 

The potential relation between early infancy antibiotic exposure and weight issues for children is a burgeoning field of investigation, with new SRMAs and a new cross-sectional study including twins published between 2018–2020. Below, we summarized the consistency and methodological aspects of recent studies including the 2 highest quality SRMAs ^38, 39^. In a SRMA conducted by Rasmussen *et al.* (2018) ^38^, 13 studies were included with 8 studies included in the meta-analysis. The reasons for excluding 5 studies from the quantitative synthesis were: high risk of bias and incomplete data for use in the meta-analysis. The outcomes of interest were childhood weight, obesity and body mass index (BMI). The study concluded that antibiotic exposure in early infancy is associated with slightly higher risk of combined overweight or obesity in childhood (OR 1.11, 95% CI 1.02 to 1.20). The study also conducted a subgroup analysis involving number of exposures to antibiotics and time point of exposure. More than 1 antibiotic treatment among participants was associated with an OR of 1.24 (95% CI 1.09 to 1.43), and exposure within the first 6 months of life was associated with an OR of 1.20 (95% CI 1.04 to1.37). Similarly, Aghaali *et al.* (2019) ^39^ conducted a systematic review and meta-analysis of 19 total studies. The outcomes of interest were childhood overweight or obesity, difference in childhood body mass index (BMI) and weight between the exposed group and the non-exposed group. The study found a significant association between early childhood antibiotic exposure (<2 years) and risk of childhood weight gain and obesity (OR 1.05, 95% CI 1.04 to 1.06). The study also conducted a subgroup analysis involving time point of exposure. Among infants exposed to antibiotics before 6 months of age, the odds of more weight gain was 11%, while for infants exposed to antibiotics after 6 months of age, the odds of more weight gain was 7%. However, unlike our review, Rasmussen *et al.* (2018) ^37^ and Aghaali *et al.* (2019) ^39^ did not conduct a test of interaction for their subgroups based on the number of exposures and timing of exposure, nor was there a study protocol available. In addition to having a publicly available protocol, our study is the only systematic review and meta-analysis to rate the quality of the evidence for each outcome using the GRADE approach, and to present data as an absolute risk difference, an approach to presenting results that has been shown to be more intuitive and helpful for decision-makers ^40, 41^.

Our findings must be interpreted in the light of a recent cross-sectional study ^42^ of 284,211 participants that included siblings and twins in New Zealand and that analyzed prenatal and early infancy antibiotic exposure (first two years) and its association with obesity at 4 years. The study found that both prenatal and early infancy exposure to antibiotics were independently associated with obesity at 4 years in a dose dependent manner. For the child’s exposure, the OR for the association between antibiotic exposure and obesity was 1.04 (95%CI 1.03 to 1.05) among siblings and 1.05 (95%CI 1.02 to 1.09) among twins. However, fixed effect analysis of siblings (6249) and twins (522) with discordant outcomes showed no association between antibiotic exposure and obesity, with ORs of 0.95 (95%CI 0.90 to 1.00) for maternal exposure, 1.02 (95%CI 0.99 to 1.04) for early infancy exposure among all children, and 0.91 (95%CI 0.81 to 1.02) for twins’ exposure. The findings by Leong *et al.* (2020) indicates unmeasured confounding factors in previous studies, and supports our findings, indicating very low quality evidence for a trivial to very small increased risk of weight issues later in life.

Overall, our systematic review suggests that prenatal and early childhood antibiotic exposure is associated with an increased associated risk of overweight and obesity among children and adolescents, potentially independent of more established early determinants of obesity. Our findings suggest that the first few years of life may represent a critical window of development, where external exposures, such as antibiotics, may program metabolic pathways and obesity risk through mechanisms involving the gut microbiota; however, experimental studies are needed to establish the impact of antibiotics, particularly frequent antibiotic use, on the gut microbiota and how this may impact weight-gain in humans. Until registered protocol-driven higher quality cohort studies with explicit plans for adjustment and statistical analysis that better demonstrate an antibiotic dose-response curve or controlled clinical trials (e.g. watchful waiting for otitis media) with long-term follow-up are conducted to confirm or refute these findings, very low quality evidence raises serious questions about the plausibility of prenatal and early antibiotic exposure being causally related to weight in children.

## Data availability

### Underlying data

All data underlying the results are available as part of the article and no additional source data are required.

### Extended data

Harvard Dataverse: Antibiotics and Weight Outcomes, Diabetes and Microbiome Literature Search. https://doi.org/10.7910/DVN/BOGPJL ^14^

This project contains the following extended data:

- antibiotics_health_outcomes_search_strategy.docx (Search Strategy)

### Reporting guidelines

Harvard Dataverse: PRISMA checklist for ‘Early and frequent exposure to antibiotics in children and the risk of obesity: systematic review and meta-analysis of observational studies’ https://doi.org/10.7910/DVN/BOGPJL ^14^
