# Supplementary material for: Early and frequent exposure to antibiotics in children and the risk of obesity: systematic review and meta-analysis of observational studies
Source: F1000Res. 2020 Jul 16;9:711. [Version 1] doi: 10.12688/f1000research.24553.1 (PMC7429923; doi:10.12688/f1000research.24553.1)
Supplement: Peer review history

# Peer review: Reviewer response for version 1

**DOI:** 10.5256/f1000research.27087.r68273

**Published:** 2020-08-13

**Authors:** Xiuxia Li

**Affiliations:** 1 School of Public Health, Lanzhou University, Lanzhou, China

The authors conducted a systematic review and meta-analysis to evaluate evidence on the impact of prenatal, intrapartum, and early childhood antibiotic exposure and the association with the risk of overweight and obesity later in childhood. They concluded that very low-quality evidence suggests that exposure to antibiotics early in life may be associated with an increased risk of overweight and obesity in later childhood. This manuscript is well-written and the reporting quality is good. However, few edits should be made before indexing.

1. The authors should report any change/differences between the registered PROSPERO protocol and published manuscript. For example, different review question, different databases searched, different inclusion criteria and so on.
2. Please keep the inclusion criteria consistency about study design in the abstract section and those in the manuscript (‘We conducted a comprehensive search of Embase, MEDLINE, and Web of Science for observational studies’ vs Inclusion criteria ‘Observational and experimental study designs were eligible’).
3. The initial records (12091+25) in the flow diagram did not match with the in the 12091 in the results section. Please modify.

Are the rationale for, and objectives of, the Systematic Review clearly stated?

Yes

Is the statistical analysis and its interpretation appropriate?

Yes

Are sufficient details of the methods and analysis provided to allow replication by others?

Yes

Are the conclusions drawn adequately supported by the results presented in the review?

Yes

Reviewer Expertise:

systematic review methodology

I confirm that I have read this submission and believe that I have an appropriate level of expertise to confirm that it is of an acceptable scientific standard.

# Peer review: Reviewer response for version 1

**DOI:** 10.5256/f1000research.27087.r67985

**Published:** 2020-08-10

**Authors:** Bartłomiej M. Zalewski, Maciej Kołodziej

**Affiliations:** 1 Department of Paediatrics, Medical University of Warsaw, Warsaw, Poland

The reviewers' objective was to evaluate evidence on the impact of prenatal, intrapartum, and early childhood antibiotic exposure and the association with the risk of overweight and obesity later in childhood.

 To answer this question, the researchers decided to include in their systematic review both experimental and observational studies, however, only observational data was identified at this time. The Reviewers concluded that very low-quality evidence suggests that exposure to antibiotics early in life may be associated with an increased risk of overweight and obesity in later childhood.

 Comments that should be addressed:

**General comments:**

 1: There are significant deviations from the PROSPERO protocol that should be commented on/explained:

- different review question,
- different databases searched (less than pre-planned),
- different inclusion criteria (i.e. Protocol: the authors stated “Participants/population: Children aged 0 to 18, of any body weight and health status will be included.”; Manuscript: the authors did study the maternal prenatal and intrapartum antibiotic exposure.)
- two of the main outcomes (diabetes and change in the microbiome) were not reported at all. (If the authors did not find any studies we would recommend adding such a statement in the manuscript),
- different methods for the risk of bias assessment - what was the rationale for this change?
- pre-planned analyses of subgroups are different in the final paper than in the PROSPERO record [i.e. pre-planned analyses not done: subgroup analyses for boys versus girls; the effect of broad vs. narrow spectrum of antibiotics will be analyzed for all outcomes; we will assess the potential impact of early exposure (less than 3 years of age) to antibiotics versus later exposure (3 years of age or greater) (but done for 7y vs >=7y)].

 

 In line with the above commentaries, the cited sentence should be changed accordingly (" *a priori* design was published as a written protocol and registered with PROSPERO ^13^. We closely followed our *a priori* protocol"). 

 2a: In the methods section, the main outcomes of interest (overweight and obesity) are defined as BMI >25 kg/m2 for overweight and >30 kg/m2 for obesity, which is typically used in adults. It should be explained why the authors did not consider (in the methods) different, more appropriate outcomes for children definitions of overweight/obesity (i.e. >1SD for overweight and >2SD for obesity in children 5-19y in accordance to WHO).

 As most of the included studies were reporting the main outcome in percentiles – therefore the methods section should be changed accordingly.

 2b: In the PROSPERO protocol, pre-specified outcomes were 1. Overweight 2. Obesity. Please clarify what was behind the decision to group the second outcome as Overweight + Obesity? Why was the pre-planned outcome of "obesity only" not reported at all? There is at least a theoretical chance that the overweight effect was big enough to make a grouped outcome of overweight + obesity also significant, even if obesity alone was non-significant (alone). It would be of interest for all readers to complement this review with analysis for the obesity outcome only (as was pre-planned).    

 3: As the literature search was conducted >24 months apart of completion of the review, and the Reviewers report other relevant studies in their discussion, one should consider updating the search, as following guidance from the Cochrane Handbook: “Reviews that are out of date and do not incorporate all the available evidence risk providing misleading information to decision makers and other stakeholders.” https://training.cochrane.org/handbook/current/chapter-iv.

**Additional specific comments:**

**Abstract: **

1. Currently, the methods in the abstract do not match with those in the manuscript – please correct: (‘We conducted a comprehensive search of Embase, MEDLINE, and Web of Science for observational studies’ vs Inclusion criteria'. Observational and experimental study designs were eligible).
2. Results section: could you add the number of participants eligible for both calculations of overweight and (overweight + obesity)?

 
**Results:**

1. The flow diagram shows that the authors identified 12116 (12091+25). However, in the manuscript there is the number 12091 – please clarify.
2. Could the authors provide the Table of Excluded studies?
3. Does the excluded studies table contain: Poulsen et al. (2017 ^1^)? If not, what was the reason to exclude this study?
4. To increase readability, numerical references should be added in Table 1 near each included study.
5. Figure 7 – not described adequately – low risk of bias?

Apart from the specific comments, we have additionally assessed this systematic review using the AMSTAR-2 critical appraisal tool (Shea *et al.*, 2017 ^2^) with an overall rating of confidence as moderate/low (due to deviations from the PROSPERO protocol and other non-critical weaknesses). "No" was answered to the following domains in the AMSTAR 2 tool: 2; 7,10;13.

Are the rationale for, and objectives of, the Systematic Review clearly stated?

Yes

Is the statistical analysis and its interpretation appropriate?

Partly

Are sufficient details of the methods and analysis provided to allow replication by others?

Yes

Are the conclusions drawn adequately supported by the results presented in the review?

Partly

Reviewer Expertise:

obesity; evidence-based medicine

We confirm that we have read this submission and believe that we have an appropriate level of expertise to confirm that it is of an acceptable scientific standard, however we have significant reservations, as outlined above.

## References

[1] : Associations of prenatal and childhood antibiotic use with child body mass index at age 3 years. *Obesity (Silver Spring)*.25(2) : 10.1002/oby.21719 438-444 10.1002/oby.21719 28124504PMC5301467

[2] : AMSTAR 2: a critical appraisal tool for systematic reviews that include randomised or non-randomised studies of healthcare interventions, or both. *BMJ*.2017; 10.1136/bmj.j4008 10.1136/bmj.j4008 PMC583336528935701

# Peer review: Reviewer response for version 1

**DOI:** 10.5256/f1000research.27087.r68274

**Published:** 2020-08-03

**Authors:** Sepideh Soltani

**Affiliations:** 1 Department of Nutrition, Faculty of Health, Shahid Sadoughi University of Medical Sciences, Yazd, Iran

The manuscript by Srivastava *et al.* examines the association between Early and frequent exposure to antibiotics in children and the risk of obesity. This is a well-conducted thorough review and meta-analysis. The literature search and the meta-analysis appear to have been conducted appropriately. The methods are well defined and the data appropriately statistically analyses. The figures and tables, including supplementary material, are appropriate. Overall the discussion is well written. There is no major concern about this manuscript.

Are the rationale for, and objectives of, the Systematic Review clearly stated?

Yes

Is the statistical analysis and its interpretation appropriate?

Yes

Are sufficient details of the methods and analysis provided to allow replication by others?

Yes

Are the conclusions drawn adequately supported by the results presented in the review?

Yes

Reviewer Expertise:

my research interest is systematic review and meta-analysis of observational and clinical trial studies.

I confirm that I have read this submission and believe that I have an appropriate level of expertise to confirm that it is of an acceptable scientific standard.
